# Exploring Models for an International Legal Agreement on the Global Antimicrobial Commons: Lessons from Climate Agreements

**DOI:** 10.1007/s10728-019-00389-3

**Published:** 2020-01-21

**Authors:** Susan Rogers Van Katwyk, Alberto Giubilini, Claas Kirchhelle, Isaac Weldon, Mark Harrison, Angela McLean, Julian Savulescu, Steven J. Hoffman

**Affiliations:** 1 Global Strategy Lab, Dahdaleh Institute for Global Health Research, Faculty of Health and Osgoode Hall Law School, York University, 4700 Keele Street, Dahdaleh Building 2120, Toronto, ON M3J 1P3 Canada; 2 School of Epidemiology and Public Health, Faculty of Medicine, University of Ottawa, Ottawa, Canada; 3 Oxford Martin School, University of Oxford, Oxford, UK; 4 Wellcome Centre for Ethics and Humanities, University of Oxford, Oxford, UK; 5 Wellcome Unit for the History of Medicine, University of Oxford, Oxford, UK; 6 Department of Politics, York University, Toronto, Canada; 7 Department of Zoology, University of Oxford, Oxford, UK; 8 Oxford Uehiro Centre for Practical Ethics, University of Oxford, Oxford, UK; 9 Department of Global Health and Population, Harvard T H Chan School of Public Health, Harvard University, Boston, MA USA; 10 Department of Health Research Methods, Evidence, and Impact and McMaster Health Forum, McMaster University, Hamilton, Canada

**Keywords:** Antimicrobial resistance, International law, Global health policy, Collective action

## Abstract

An international legal agreement governing the global antimicrobial commons would represent the strongest commitment mechanism for achieving collective action on antimicrobial resistance (AMR). Since AMR has important similarities to climate change—both are common pool resource challenges that require massive, long-term political commitments—the first article in this special issue draws lessons from various climate agreements that could be applicable for developing a grand bargain on AMR. We consider the similarities and differences between the Paris Climate Agreement and current governance structures for AMR, and identify the merits and challenges associated with different international forums for developing a long-term international agreement on AMR. To be effective, fair, and feasible, an enduring legal agreement on AMR will require a combination of universal, differentiated, and individualized requirements, nationally determined contributions that are regularly reviewed and ratcheted up in level of ambition, a regular independent scientific stocktake to support evidence informed policymaking, and a concrete global goal to rally support.

## Introduction

International legal agreements are formally the strongest mechanisms through which countries can make commitments to each other [15], but often require significant political mobilization and are rare in global health. In the face of a serious threat to global public health, however, an international legal agreement provides a regulatory framework that can bind countries together, provide accountability for turning commitments into action, and disincentivize countries from breaking their promises.

Antimicrobial resistance (AMR) is one of the leading threats to global health and sustainable development [35]. The threat posed by AMR has been compared in magnitude to that posed by climate change, and the two collective action problems have many similarities [14]. There is a risk that resistance will develop every time a microbe is exposed to an antimicrobial agent. This vulnerability makes the effectiveness of antimicrobials—one of the pillars of modern medicine—a global common-pool resource that must be collectively managed. Yet AMR is not unique in that it has similar problem features to other global common-pool resource challenges. Like climate change, AMR is a natural process accelerated by humans’ mass introduction of antimicrobial substances into the environment, structural inequalities that impede access to effective infection prevention and control in resource limited settings, market failures that disincentivize technological developments, coordination failures that prevent alignment across implicated sectors, and governance failures that discourages countries from acting on this problem unless others do so as well. Without regulations upholding the common good, the world risks depleting or spoiling the common pool of antimicrobial effectiveness, as individual actors will naturally seek to address their own needs in the short-term, rather than preserving the long-term effectiveness of antimicrobials for everyone. Thus, antimicrobial consumption is a standard case of “the tragedy of the commons”. From a biological perspective, past attempts to curb AMR have failed because of the historically uninterrupted rise of global antimicrobial use and subsequent proliferation of resistance conferring genes [21]. From a political and social perspective, past attempts to tackle AMR have failed because stewardship regimes have been too narrow in national or sectoral scope, and policy responses at the international level have been fragmented [17, 19].

The historical failure of country-level and voluntary solutions means that binding international legal agreements may have a stronger likelihood of protecting the global antimicrobial commons. A global health treaty has a reasonable chance of being impactful if it meets four criteria: first, there must be a significant transnational dimension to the health challenge; second, the treaty must address multilateral challenges that cannot practically be addressed by a single country and therefore justifies the use of an instrument with coercive features; third, the proposed treaty should have a reasonable chance of achieving benefits by incentivizing action, institutionalizing accountability, and activating interest groups; and fourth, the treaty should represent the best commitment mechanism among the competing alternative strategies [15]. Despite calls for various international legal agreements on health issues, only two health-specific international legal agreements currently exist (i.e., the International Health Regulations and the Framework Convention on Tobacco Control). A global treaty on AMR merits serious consideration as AMR is one of the few threats where a global treaty could satisfy these four requirements.

Although examples of health-specific international legal agreements are scarce, recent international climate change agreements offer a useful comparator, since both climate change and AMR require collective action to address problems that resemble a global tragedy-of-the-commons. In this article, we look to recent international climate change agreements to identify design elements that might inform the development of a microbially effective, ethically fair, and politically feasible AMR treaty.

## Three Different Models from Past Agreements

Past environmental agreements offer at least three ways of delineating state parties’ responsibilities (Table 1). In the first model—*universal responsibilities* or “one-size-fits-all”—every country is bound by the same targets and obligations for action. This model was used by many environmental treaties prior to the 1990s [11], however, the key limitation of the universal responsibilities model was that it did not account for the fact that every country contributed to the problem in a unique way and could contribute differently to the solution. Indeed, different levels of liability imply different ethical obligations to solve the problem. Imposing equal responsibilities on all countries could prove triply unfair in relation to relative contributions to the problem, relative costs of responding, and relative benefits reaped. In the case of AMR, depending solely on a universal responsibilities model would likely be counterproductive, as an ethically fair AMR agreement would feature different targets and obligations for different countries according to their contribution to the problem (e.g., historical rates of antimicrobial use) and capacity to respond (e.g., economic status).

**Table 1:** Three models for global treaty design

Model	Universal responsibilities	Differentiated responsibilities	Individualized responsibilities
Description	One set of obligations for all countries	Different sets of obligations for HICs and LMICs	Countries individually determine their own obligations
Potential strengths	Equality among all countries	More equitable as it considers differing abilities of HICs and LMICs	Potentially most equitable model if implemented in good faith and fairly
Critical limitation for an AMR agreement	Equity concerns and impossibility for many countries to meet a single universal standard	Division into two camps masks the ways countries contribute to AMR and can address it	No common AMR goal against which to assess individual commitments and the difficulty of allocating AMR responsibility

The second model of crafting states parties’ responsibilities—*differentiated responsibilities*—sets different standards for different groups of countries, usually one standard for high-income countries (HICs) and another for LMICs. The 1997 Kyoto Protocol to the United Nations (UN) Framework Convention on Climate Change (FCCC), for instance, set collective emission-reduction targets for HICs and different requirements for LMICs. Given the overconsumption of antimicrobials in HICs, and the need to focus on boosting access to life-saving drugs in many LMICs, some scholars have suggested that a differentiated responsibility model should be applied to AMR (e.g. [20]). However, the overuse of antimicrobials is not driven solely by economic status. Disease burden, regulatory capacity, and a myriad of health system and societal factors also contribute to the use of antimicrobials [2, 4, 10, 27]. The differentiated responsibility model by itself is therefore unlikely to be either an effective or fair model for a global AMR agreement.

The third model—*individualized responsibilities*—is embodied by the 2015 Paris Agreement under the UN FCCC. In the Paris model, participating parties autonomously commit to individualized targets and actions based on feasibility determined at the national level and informed by a common global goal. This approach enables a bespoke distribution of responsibilities for action which, if implemented in good faith, could be fair and effective. While every aspect of international legal agreements cannot be entirely individualized, these kinds of responsibilities give countries maximum flexibility to self-determine how they act towards a clear collective goal like, in the case of the Paris Agreement, keeping global average temperatures well below 2 °C above pre-industrial levels. Each country’s individual commitments are declared publicly in a national action plan, subjected to review by a centralized authority [3], and the global target provides a criterion for assessing whether these pledges represent fair and effective contributions. Notwithstanding the current lack of an analogous global target, an AMR treaty could emulate this model by elaborating a fair and effective distribution of responsibilities based on individual countries’ contributions to AMR as well as their individual capacities to implement effective conservation policies without compromising public health.

## The Paris Agreement as a Model for Action

The Paris Climate Agreement, which overcame many limitations of previous climate change agreements, can serve as an equitable and fair agreement model and presents a set of useful lessons for international law-making in the AMR context. We have identified seven essentialized design elements of the Paris Agreement that can serve as a starting point for discussing potential elements of an international legal agreement on AMR: (1) a collective global goal; (2) nationally determined contributions that are pledged, reviewed and ratcheted every 5 years; (3) regular reporting on actions and outcomes; (4) an annual conference of parties; (5) a global scientific stocktake every 5 years; (6) a combination of binding obligations and recommended policies; and (7) an international legal framework.

Developing these elements from a blank slate would likely be very difficult. However, some similar elements already exist in global AMR governance. A future international legal agreement on AMR can therefore draw upon many existing elements for momentum, build upon existing institutional structures, and coordinate efforts to reinforce existing action on AMR (Table 2).

**Table 2:** Comparing essential elements of the Paris Agreement with existing global AMR efforts

Essential elements	Paris Agreement	Global AMR efforts
(1) Collective global goal	Keep global temperature rise below 2 °C above pre-industrial levels	*No consensus on what a common global goal could look like.*
(2) Nationally determined contributions pledged, reviewed and ratcheted every 5 years	All parties must communicate their nationally determined contributions every five years, and, during revisions, aim for maximally ambitious goals. Nationally determined contributions are reviewed to ensure the distribution of responsibilities is fair and that countries are ambitious in their goals	All WHO member states committed to have national action plans for AMR. Even though this commitment is not legally binding, over 100 have such plans and many are under development *No review process or ratcheting mechanism*
(3) Regular reporting on activities and outcomes	All parties must regularly provide information on activities and outcomes, using methods that are articulated by the Intergovernmental Panel on Climate Change	WHO, FAO, OIE conduct self-assessment surveys on countries’ AMR activities. *No regular reporting of outcomes* *No standard methodology for reporting outcomes*
(4) Annual Conference of Parties	The UNFCCC’s Annual Conference of the Parties serves as the conference of parties for the Paris Agreement	AMR is normally discussed every 3 years at the World Health Assembly *No formal or regular meeting*
(5) Global scientific stocktake every 5 years	Requirement to take stock of the best available science every 5 years. This stocktaking exercise will help ensure that the agreement’s ongoing efforts are in line with scientific best practices	*No relevant comparison*
(6) Combination of binding obligations and recommended policies	Legally binding obligations are mostly procedural, but include some mandatory activities. The Paris Agreement makes recommendations for countries to maximize their contributions	The WHO, FAO, and OIE maintain a list of recommended policies for countries to include in their national action plans *Nothing legally binding*
(7) International legal framework	The Paris Agreement is a legally binding instrument of the UNFCCC. The UNFCCC provides a broader legal framework for the Paris Agreement	*No international legal framework, although the constituting instruments of the WHO, FAO, OIE or UN could serve as the broader legal framework for a legally binding AMR agreement*

1. A collective global goal

The Paris Agreement codified an overarching goal to keep global warming well below 2 °C compared to pre-industrial levels. This collective goal provides common ground for negotiation, collaboration, and national policymaking. Developing an equally clear and feasible target would be helpful for progress on AMR; however, there is currently no consensus on an analogous, quantifiable goal for AMR containment. Developing such a goal is likely to be the first, and perhaps most important, challenge in developing an international legal agreement on AMR. Numerous AMR indicators have been proposed, and, in a positive development, a new indicator for antimicrobial resistant blood stream infections has been proposed to support the Sustainable Development Goals [23]. The international community would still benefit from a broad political goal to support negotiation, collaboration, and national policy making, however, setting such a goal will prove difficult considering there will never be one ‘safe’ level of global antimicrobial use and the necessity of balancing access, conservation, and innovation across human, animal, agricultural and environmental sectors.

(2) Nationally determined contributions that are pledged, reviewed and ratcheted every 5 years

Perhaps the most unique design element of the Paris Agreement is the universal responsibility imposed on all countries to articulate their own individualized responsibilities in national action plans. In this system, nationally determined contributions are pledged and then subsequently reviewed and ratcheted-up in level of ambition every 5 years. The requirement that each successive round of pledged contributions must represent a progression beyond earlier contributions ensures that plans are updated in accordance with increasing response capacity.

The global AMR regime includes the foundation for a similar approach. In 2015, WHO’s plenary governing body—the World Health Assembly—called upon all countries to develop national action plans to address the threat of AMR by 2017 [33]. In total, 100 countries met this goal and many others have national action plans in development [36]. Having countries determine their own action plans is an ideal strategy for tackling AMR because it means that countries can tailor specific goals to fit local challenges and health system contexts, while preserving, more broadly, principles of effectiveness, fairness, and feasibility [29]. Current global governance of the antimicrobial commons leaves much room for improvement. At present, there is no requirement for countries to update their plans or gradually increase the level of ambition therein, nor was the initial request from the World Health Assembly legally binding. The pledge, review, and ratchet mechanism present in the Paris Agreement is therefore a potentially useful option for building on existing AMR efforts and increasing countries’ levels of ambition and action over time.

Yet, if developing global consensus around a goal is the first major challenge, spurring national action to reach this goal is the second. Initial country pledges under the Paris Agreement were insufficient to meet the global target established in that agreement, and similarly, national AMR action plans have to date been insufficiently ambitious. Although most high antimicrobial-using countries have made public commitments to reduce their use of these products, it has thus far proved difficult to translate this engagement into sufficient national or global policy action [36].

(3) Regular reporting on activities and outcomes

Parties to the Paris Agreement are legally bound to regularly report on both activities and outcomes related to their commitments using a standard and accepted methodology. In addition to regular reporting, countries are legally obligated to participate in verification processes to ensure high-quality data reporting. While these mechanisms have not yet been activated, from a design point of view, the robust monitoring, reporting and verification mechanisms of the Paris Agreement may provide some useful lessons for overcoming current weaknesses in global AMR monitoring and reporting processes. The tripartite coalition of WHO, FAO, and OIE currently monitors national-level progress in developing and implementing their national action plans through self-assessment surveys; however, these surveys currently lack an effective verification system [36]. Mechanisms to monitor outcomes are even less developed, as many countries lack surveillance systems capable of monitoring antimicrobial usage or AMR. The WHO has established a Global Antimicrobial Resistance Surveillance System (GLASS) to address this gap, but despite its best effort to standardize AMR monitoring, GLASS still cannot harmonize surveillance efforts as there are no universally accepted metrics or methodologies for measuring AMR across disciplines and organizations and the system suffers from poor enrollment [34].

(4) An annual conference of parties

The Paris Agreement reaffirms countries’ previous commitments to meeting annually to discuss their progress, chart further actions, continue working on existing challenges, and address new issues that emerged in the preceding year. These regular meetings enhance cooperation by building trust, offering predictability, and sharing knowledge and best practices—each a valuable aspect from which the AMR regime could benefit. Over the past 80 years, AMR has not maintained a long-term presence on the global health agenda. The closest AMR has come to achieving this kind of sustained attention is the World Health Assembly’s informal custom of revisiting this major health issue roughly every 3 years. The potentially catastrophic loss of life and the devastating economic impacts associated with AMR should merit more frequent ongoing, high-level discussions of the kind mandated by the Paris Agreement on an annual basis.

(5) A global scientific stocktake every 5 years

In addition to annual meetings, countries also agreed to participate in a science-based “global stocktake” every 5 years for the purpose of assessing progress towards the Paris Agreement’s long-term goals. This stocktake also features a review of relevant scientific evidence, which allows global climate action to evolve as new scientific discoveries come to light and supports countries to make evidence-informed climate policies. No similar mechanism exists to inform AMR policy, yet an AMR agreement could include this stocktake mechanism to support evidence-informed policy. Currently, there is little evidence on the relative effectiveness of AMR interventions, existing evidence is not often used to inform policy action [28], and there is no consensus on what constitutes good evidence for AMR policy. A global stocktake of scientific evidence to inform AMR policymaking would emphasize the importance, and ultimately support the production, of high-quality scientific evidence on both the state of the problem posed by AMR and the solutions available to countries for their potential implementation.

(6) A combination of binding obligations and recommended policies

The Paris Agreement achieved broad support by combining legally binding and non-binding provisions. Major carbon emitters, who were reluctant to commit to a rigid set of predetermined emissions reductions, were open to a treaty that combined legal requirements and recommendations [7]. Legal requirements in the Paris Agreement include the development of nationally determined contributions and the introduction of adequate reporting and review mechanisms. The specific content of contributions was not mandated but, instead, is to be determined by each country through its own domestic processes in a way that reflects each country’s abilities. In theory, the mechanism that drives states to continually commit to ambitious action is a form of soft reciprocity among states, meaning it depends on a culture of solidarity, trust, and good faith. Some states take the lead and act towards solving the collective problem, trusting that other states will reciprocate by doing their part in the future. Countries are expected to show global solidarity and are likely to experience negative reactions from other countries and suffer reputational consequences if they do not participate in good faith or fail to live up to their commitments. One obstacle to achieving this type of culture for AMR is that most (if not all) states would have to acknowledge the necessity of acting on the threat of AMR—a requirement that could run up against other local health priorities. Nonetheless, the same hybrid mechanism might be applied in the case of an AMR agreement, including building and revising national action plans, setting standards and requirements for surveillance, data collection and reporting, and providing financial resources to LMICs to implement the agreement’s provisions.

(7) An international legal framework

The Paris Agreement, as a binding instrument within the UN FCCC, provides a relatively comprehensive legal framework for climate change action. Despite the predicted suitability of an international legal framework governing the antimicrobial commons, there does not exist a binding legal instrument to hold parties accountable for antimicrobial stewardship. Having been negotiated to overcome a similar global tragedy-of-the-commons challenge, adopting a framework similar to that of the Paris Agreement may provide a robust and adaptable framework for developing and sustaining political commitment to effectively, fairly and feasibly manage the global antimicrobial commons. Yet, the flexibility offered to countries in meeting their individualized responsibilities might also hinder the achievement of the collective goal, and, as previously noted, initial country pledges under the Paris Agreement were insufficient to meet the global target. As such, some incentives or penalties might be desirable in an AMR agreement in order to promote compliance with countries’ targets. Collective action problems that require cooperation of a large number of parties sometimes suffer from a ‘problem of assurance.’ The problem of assurance can arise when individuals are sincere in their willingness to contribute, but skeptical of the sincerity of other parties. When costs of defection are low and benefits non-excludable, as in free-rider-prone issues like AMR, the assurance problem is more likely to arise. If it does, then parties will likely only be willing to contribute if they have the assurance that others are making their fair contribution as well. If countries were legally bound and incentivized to meet their own targets in terms of ensuring access, conservation and innovation for AMR, this could increase the chances of success for an AMR agreement.

## AMR Treaty Provisions

To protect the global antimicrobial commons, an effective international legal framework will need to comprise a mix of universal, differentiated and individualized requirements. In creating a global AMR treaty, it is essential to acknowledge that the provisions discussed below will have important implications for gender equity, intergenerational equity, the empowerment of women, the right to health, and the rights of various marginalized groups. At a minimum, any AMR treaty should include language similar to the Paris Agreement that recognizes these essential factors and requires countries to respect and account for this diversity.

### Universal Requirements

Many universal requirements of an AMR treaty could mirror the universal requirements of the Paris Agreement; for example, a universal requirement to regularly report AMR related activities and outcomes, or a requirement to pledge, review, and ratchet nationally determined contributions. Other universal treaty requirements could support these activities; for example, establishing universal AMR and antimicrobial usage definitions, compulsory and transparent monitoring standards, and other requirements to improve data collection would support surveillance systems and the development of long-term national action plans. Historically, one of the greatest hurdles for global antimicrobial stewardship has been the lack of internationally standardized and transparent data collection on antimicrobial usage and AMR. The US, EU, and Asian HICs still vary in their approach to AMR and antimicrobial usage definitions, measurement methods, and data processing, and it has proved difficult to reconcile data collected through active monitoring protocols in human medicine with passive monitoring systems often employed in non-human settings. Despite launching GLASS, international organizations like WHO and OIE have faced some unwillingness among countries to openly and transparently report their AMR and antimicrobial usage data [31, 34].

### Differentiated Requirements

Disease burdens vary significantly between LMICs and HICs, access to antimicrobials is still limited in many parts of the world, and not every country has the economic, bureaucratic, and scientific resources to implement stewardship requirements. Meanwhile, higher levels of per capita consumption and their historical role in spreading antimicrobial consumption mean that HICs have a greater moral obligation to shoulder greater burdens when it comes to tackling the resulting global fallout of AMR [18, 20]. As a result, in addition to universal requirements, any international legal framework aiming to effectively, fairly and feasibly manage the global antimicrobial commons must also include differentiated requirements.

An international treaty can acknowledge this differentiated responsibility by requiring HICs to make greater or earlier reductions in human, animal, and environmental antimicrobial usage and other targets (e.g., surveillance capacity) as outlined in the treaty’s overarching goal. Furthermore, differentiated requirements might require HICs to finance the development of new antimicrobials, diagnostics, and alternative treatments, or by requiring HIC contributions to an international fund designed to support the integrated development of health care, food, and water, sanitation, and hygiene (WASH) systems in LMICs. Differentiated responsibility is already informing the financing of delinked antibiotic development through public–private partnerships (e.g., CARB-X) by countries like the US, UK, and Germany. However, overall funding remains limited and will not be indefinite. New investments could be required either via existing public–private partnerships such as the Global Antibiotic Research and Development Partnership (GARDP) or via a new international body for antimicrobial development [30].

### Individualized Requirements

Like the Paris Agreement, an international legal framework on the global antimicrobial commons can also include a process requirement for countries to make individualized commitments towards achieving a common goal. While the requirement to outline nationally determined contributions is universal, domestic mitigation measures addressing the specific contributions proposed by individual countries become legally binding upon only them. Individualized commitments on the part of countries could range from enhanced environmental and stewardship efforts by antimicrobial-producing countries to a restructuring of prescription incentives for health professionals in HICs. A recent systematic review and evidence map identified 17 distinct policy strategies that have been used by national governments to reduce human antimicrobial use [29]—any of which could potentially be included in individualized national commitments. There are also many policy strategies available to reduce animal usage of antimicrobials. For example, in many countries, veterinarians make a financial profit from prescribing and selling antimicrobials to farmers, whereas other countries have banned this practice [16]. Similarly, some countries allow veterinarians to prescribe antimicrobials to animals even though these drugs have only been licensed for human usage; drugs prescribed according to this cascade system are often not captured by official sales data [6]. Individualized legally binding commitments by counties with unique circumstances could significantly enhance the transparency and efficacy of stewardship policies while taking a tailored approach to limiting conflicts of interest in the veterinary and medical professions.

## Goal-Setting

A clear and focused goal is integral to the success of any international agreement, or indeed, any successful public health campaign. To date, the lack of such a goal in the global AMR response has proved problematic both for catalyzing action and measuring success. International goal-setting can take one of two forms. The first, as in the case of the Paris Agreement, is a specific overarching global goal. The second, as in the case of the Sustainable Development Goals (SDGs), are general goals supported by multiple specific targets. Either approach can be effective, provided it is supported by concrete, measurable indicators. A lack of generally accepted and measurable targets has been a challenge in AMR with respect to the general goal set in the 2015 WHO Global Action Plan on Antimicrobial resistance, “To ensure, for as long as possible, continuity of successful treatment and prevention of infectious diseases with effective and safe medicines that are quality-assured, used in a responsible way, and accessible to all who need them,” which lacks the level of specificity needed to be actionable or to measure progress towards globally agreed priorities.

The examples from the Paris Agreement and the SDGs also illustrate the three approaches to goal setting: directional goals, absolute goals, and relative goals. The SDG target 3.3 to “end the epidemic of AIDS, tuberculosis, malaria and neglected tropical diseases…” is an *absolute goal*, for which there is a strict definition of success. In the case of AMR, an absolute goal might be to end all deaths from amenable infections or to end the use of critically important drugs in non-human sectors by 2030. The Paris Agreement goal, and the SDG target 3.4 to “reduce by one-third premature mortality from non-communicable diseases…”, are both examples of *relative goals* which have specific targets defined in relation to other measures. A relative goal in the case of AMR might be to reduce deaths from drug-resistant infections overall or to reduce the rate of specific drug-resistant infections (e.g., methicillin resistant Staphylococcus aureus) below a designated reference year. Finally, SDG target 3.9 to “substantially reduce the number of deaths and illnesses from hazardous chemicals…” provides an example of a *directional goal* which focus on progress from a starting place without setting a clear benchmark. In the case of AMR, this might include a goal to minimize deaths from preventable infections—which depends on both achieving greater access to antimicrobials and conserving the effectiveness of existing antimicrobials. Achieving a microbially effective, internationally equitable, and politically feasible grand bargain on AMR will require a substantial effort to clearly define AMR priorities in terms of either a single specific and measurable goal, or a broad commitment to reducing AMR through an agreed series of specific AMR targets. At the very least, the goal(s) for AMR will need to address both the access and conservation dimensions of the AMR problem. Two preliminary options for potential AMR goals are presented in Table 3.

**Table 3:** Approaches to international goal-setting, SDG 3 examples, and two stylized options for AMR

	SDG 3 Example	Option 1 for AMR	Option 2 for AMR
Absolute goal	SDG target 3.3 to end the epidemic of AIDS, tuberculosis, malaria and neglected tropical diseases…	Zero deaths from susceptible infections (which is a goal for antimicrobial access)	–
Relative goal	SDG target 3.4 to reduce by one-third premature mortality from non-communicable diseases…	Reduce deaths from drug-resistant infections below 2015 levels (which is a goal for antimicrobial conservation)	–
Directional goal	SDG target 3.9 to substantially reduce the number of deaths and illnesses from hazardous chemicals…	–	Minimize deaths from preventable infections (which is a goal that balances antimicrobial access and conservation)

## Convening Forums

A legal agreement could potentially be negotiated through any of four forums, including (1) the UN General Assembly, (2) one of the UN’s relevant technical agencies or a treaty body, (3) a partnership of several UN’s agencies or treaty bodies, or (4) as a plurilateral agreement with a small group of countries. Each of these options presents certain strengths and limitations, and each has its own unique strategic considerations (Table 4).

**Table 4:** Potential forums for an AMR treaty

Forum	Strengths	Potential limitations	Strategic considerations
UNGA	High profile Higher visibility and political mobilization	Fragmentation Duplication Inefficiencies	Difficult to achieve consensus Potentially unambitious content
WHO, FAO or OIE	Technical expertise WHO Article 21 is automatically binding	Single sector engagement Relatively weak enforcement mechanisms	2/3 vote may be difficult to attain Invoking WHO’s Article 21 or revising IHR may not be popular
Joint enterprise of several agencies or bodies	Combine technical expertise across sectors Potentially multisectoral	Complicated Difficulties in negotiating responsibilities across sectors	Difficult to achieve consensus Potentially unambitious content Uncharted territory
Plurilateral agreement	Potential for maximally ambitious goals	Long-run sustainability and effectiveness uncertain Legitimacy concerns	Could generate momentum for broader participation

### UN General Assembly

The first potential forum for an international legal agreement on AMR is the UN General Assembly (UNGA). As the primary deliberative, policymaking, and representative body of the UN, UNGA would provide a high-profile forum for an AMR agreement. Such a high-profile agreement could mobilize political will and promote AMR to a higher position on national and international agendas. The UNGA’s high level of legitimacy could also facilitate collaboration among other UN and treaty bodies, including the tripartite of WHO, FAO, OIE, plus the UN Environment Program (UNEP). However, working through the UNGA would require AMR to contend for attention among other high-level security, economic and development issues, and when it gets that attention, it could suffer from a ‘race to the bottom’ in terms of consensus-building in such a large decision-making body. A treaty emerging out of UNGA would also have to properly account for, manage, and integrate ongoing AMR efforts: an overlap of authority could lead to loopholes for accountability; duplication of ongoing work could result in inefficiencies; and omissions of ongoing efforts could result in fragmentation [25].

### Technical Agency or Treaty Body

An international legal agreement could also be created under the authority of one of the UN’s relevant technical agencies. Both WHO and FAO have constitutional mechanisms permitting their members to enact legally binding rules through their plenary decision-making bodies. In fact, WHO’s constitution envisions two such mechanisms (Table 5). For example, Article 21 of the WHO constitution allows the World Health Assembly to adopt new regulations (or revise existing regulations like the International Health Regulations) that become automatically legally binding on all member states unless they proactively opt-out [32]. Adopting an AMR agreement under Article 21, however, would require interpreting this agreement as falling within the scope in which such instruments are allowed, the most relevant of which is “other procedures designed to prevent the international spread of disease”; this could potentially limit the scope of an AMR agreement. Alternatively, under Article 19 of the WHO constitution, the World Health Assembly could adopt a treaty on AMR that would become legally binding if approved by a two-thirds majority vote and subsequent adoption by participating states through their own domestic processes. Achieving the political will to take these measures may prove difficult as member states have historically avoided crafting legally binding regulations through WHO, opting instead to use the World Health Assembly for developing non-binding norms and to rely on other forums like the World Trade Organization when binding norms are needed (e.g., for regulating access to medicines). Members states have also never incorporated compliance-inducing mechanisms into WHO instruments—like incentives or penalties—which is only a limitation if countries are unwilling to live-up to their commitments [25].

**Table 5:** Mechanisms for convening a treaty under the authority of a technical agency

	FAO	WHO	
Constitutional provision	Article 14	Article 19	Article 21
Required vote	2/3 supermajority	2/3 supermajority	1/2 majority
Applies to	Members that ratify it	Members that ratify it	All members
Becomes binding	After ratification	After ratification	Automatically
Potential scope	FAO’s mandate	WHO’s mandate	“Procedures designed to prevent the international spread of disease”

WHO’s primary focus is on promoting human health, but equally important for AMR are animal health, agriculture and the environment. Countries could regulate antimicrobial use in animal and agricultural sectors using Article 14 of the FAO’s constitution, which permits members to adopt and implement legally binding conventions contingent on a 2/3 majority vote. Member states have used this FAO tool to adopt 17 conventions governing diverse issues including the regulation and promotion of livestock farming guided by principles of sustainable development and self-reliance in Asia and the Pacific. An international legal agreement on AMR under the aegis of the FAO could follow a similar model by creating a commission to regulate and promote livestock farming around the world according to the principles of AMR stewardship and conservation [8, 9]. Alternatively, the constituting instrument of the OIE—a treaty body—could be revised to create similar responsibilities and mechanisms.

### Joint Enterprise of Several Agencies or Bodies

An agreement through one of the WHO, FAO or OIE alone, however, would only address one dimension of the problem. A microbially effective response will adopt a One Health approach to bring together stakeholders across the different sectors of human, animal, agriculture and environmental health. Therefore, while either the WHO, FAO or OIE could oversee an AMR agreement, these efforts would need to be supplemented by a multisectoral partnership to include solutions that address all facets of the problem simultaneously [25]. The UN’s WHO-FAO-OIE tripartite collaboration on AMR (plus UNEP) could be the solution to overcoming this last hurdle. The tripartite brings together technical expertise and can mobilize political will and action across the different sectors. Although the tripartite does not itself have a mechanism to enact a legally binding agreement, it can act as a key governance and organizational body that catalyzes action through each of its respect mechanisms and provides critical technical support to an international legal agreement [26]. An arrangement could resemble how the UN Single Convention on Narcotic Drugs delegates some authority to WHO by requiring that all changes to the list of narcotic substances be made upon recommendations of WHO [25]. This approach could present a potential bridge between the benefits of the UNGA approach with the benefits of the technical agencies, drawing upon the tripartite’s technical expertise and current institutional momentum by integrating its resources into the adopted legal framework.

### Plurilateral Agreement

Another feasible approach might be to organize a treaty separately from any existing international organization. An initial core-group of leading countries could, most expediently, convene a meeting to begin discussions of an international treaty. While all countries must play a role in the global response to AMR, starting negotiations of an international AMR treaty with a small group of countries might generate momentum, enable more ambitious content, and allow other countries to join when they are ready [27]. A plurilateral agreement adopted by a small group may lack the legitimacy of a formal institution, but an initial small membership means that countries can more easily commit to ambitious goals, since there would be less need to lower goals to reach a consensus. In fact, this may be an appropriate approach, considering that some countries are currently in positions to act, while other are not. However, it is essential that any plurilateral agreement be open, and ready to include and confront the issues important to newly joining member states. While a closed group like the G20 might more easily come to agreement on particular aspects of AMR, such a group by itself would likely be incapable of implementing globally effective, fair, and feasible AMR governance. Moreover, the long-run equitability, sustainability and effectiveness of this approach would depend how appropriate the setup of the international agreement is for the needs of the broader global community, and on the rest of the international community’s subsequent buy-in [25, 27].

## Reaching a Win–Win–Win

Global health policies are successful when they are effective, sustainable, fair, and feasible. These criteria must be judged on a global scale; an AMR treaty cannot be deemed effective if it is effective only in certain parts of the world, and it cannot be deemed fair if it is not fair within and across countries. Finally, regardless of effectiveness, a treaty cannot be considered successful if some countries are unfairly burdened by the measures adopted. There are no cost-free solutions to AMR and some countries will have to bear a greater share of these burdens than others. The question is therefore, how can we maximize the results, minimize the burdens, distribute them fairly, and make sure everyone wins?

First, a successful treaty must strike the right balance between access, conservation, and innovation, three conflicting elements that must be addressed to satisfactorily and fairly contain AMR. Conservation compels us to reduce antimicrobial use by reducing reliance on antimicrobials. Improving hygiene standards and implementing effective infection prevention and control measures are integral strategies to achieving this goal, and investments are needed to improve health system capacity and quality of care in resource limited settings. Because the burdens need to be distributed fairly, and because the effectiveness of these measures would benefit both these countries’ populations and the global community more broadly, these investments need to be technically and financially supported by HICs. Supporting these actions would allow HICs to discharge part of their moral obligations to contribute to the solution; this would be an obligation of fairness both because HICs have historically overconsumed antimicrobials [20] and because they would benefit from the implementation of these measures. A individualized treaty model akin to the Paris Agreement would allow for tailoring these measures to individual countries and contexts, taking into account the responsibility of each country for the emergency, the capability of each country to make a contribution to solving the problem in different ways (e.g., by implementing more effective public health measures and/or by directing internal resources to other countries to assist them in this improvement), and the needs of each country. The individualized model allows for case-by-case needs assessments which will ensure that no countries are excluded from efforts to improve global health.

Second, for all countries to win, measures must be appropriate across the human health, animal, agriculture and environmental sectors. A successful treaty will give due consideration to the health needs of humans and animals and ensure that the burdens associated with promoting both are spread fairly across the actors who have so far benefited more from antimicrobials and who are more responsible for AMR. The large-scale use of antibiotics in industrial agriculture is a significant factor for the selection of AMR in the environment and animal populations [18, 24]. Within an international legal framework on the global antimicrobial commons, countries might have to implement measures to drastically reduce such consumption, although the necessary measures are likely to depend on local context, health and agricultural systems, and antimicrobial consumption. While such measures are ethically required and feasible, they necessitate imposing sacrifices on consumers and producers. The EU has already prohibited use of antibiotics for growth promotion in 2006 and decided to ban prophylactic antibiotic in 2018, but more drastic measures might be needed; for example, a Pigouvian tax on animal products obtained with the use of antimicrobials could be helpful in some contexts [13]. More generally, individual countries’ commitments to achieving their goals would require them to rethink the way intensification of farming is currently regulated, if necessary, by incentivizing certain behaviours and disincentivizing others. An increase in the costs of animal products in HICs is almost unavoidable, but the current rate of consumption and the low cost of animal products on the market is not sustainable from the point of view of human health, animal wellbeing, and the environment. Those who have so far benefited from large availability of products at low cost are ethically more responsible for the problem and it is fair that they bear a disproportionate burden to address AMR. Using incentives and disincentives appropriately based on local context would ensure that both human and animal health are promoted in synergy and with a fair distribution of burdens.

Third, an international legal agreement on the antimicrobial commons would need to achieve intergenerational justice in a way that ensures wins for both current and future generations. As with many other global problems—such as climate change—justice requires that the next generations will not have to bear too many of the burdens of the poor choices of the current generation, and that the benefits of scarce resources will last as long as possible. The benefits and burdens of antimicrobials and antimicrobial policies will need to be shared across time. But future generations will have to bear some burdens, as strategies to address the problem on the two previous dimensions will need to be constantly revised and updated, based on an assessment of the progress being made. Keeping AMR under control is a problem humanity will always face. Any treaty will need to have mechanisms in place to ensure that progress is tracked and strategies adjusted on a regular basis, so that at any time a decision can be made on how to achieve a fair distribution of the burdens, not only across countries and across sectors, but also across generations. A treaty like this will have to work over a possibly indefinite period of time so its adaptation mechanisms are essential to guarantee not only effectiveness, fairness and feasibility, but also sustainability.

## Conclusion

Antimicrobial resistance is a complex and far-reaching threat to human health and development. In this manuscript we focus on what can be learned from the design of the Paris Agreement, which complements our efforts to look at other global policy solutions for AMR [1, 5, 12, 22, 27, 33]. Although international legal agreements are rare in global health, international law represents the strongest formal mechanism for achieving global collective action in the face of pressing international threats. As two common-pool resource challenges, the similarities between AMR and climate change make the Paris Agreement a useful example, from which we can identify design elements that would facilitate the development of an effective, fair, and feasible agreement on AMR. If negotiating an AMR treaty as a joint enterprise of different international organizations proves unmanageable, then a plurilateral agreement with a small initial group of countries may offer the most feasible solution moving forward to sustainably balance access, conservation and innovation across human, animal and environmental sectors.
